# Validity of COPD diagnoses reported through nationwide health insurance systems in the People’s Republic of China

**DOI:** 10.2147/COPD.S100736

**Published:** 2016-03-01

**Authors:** Om P Kurmi, Julien Vaucher, Dan Xiao, Michael V Holmes, Yu Guo, Kourtney J Davis, Chen Wang, Haiyan Qin, Iain Turnbull, Peng Peng, Zheng Bian, Robert Clarke, Liming Li, Yiping Chen, Zhengming Chen

**Affiliations:** 1 Clinical Trial Service Unit & Epidemiological Studies Unit (CTSU), Nuffield Department of Population Health, University of Oxford, Oxford, UK; 2 Tobacco Medicine and Tobacco Cessation Center, China–Japan Friendship Hospital, Beijing, People’s Republic of China; 3 National Coordinating Centre for China Kadoorie Biobank Study, Chinese Academy of Medical Sciences, Beijing, People’s Republic of China; 4 Worldwide Epidemiology, GlaxoSmithKline R&D, Collegeville, PA, USA; 5 National Clinical Research Center for Respiratory Diseases, China-Japan Friendship Hospital, Beijing, People’s Republic of China; 6 Medical Research Center, Beijing Chao-Yang Hospital, Capital Medical University, Beijing, People’s Republic of China; 7 Radiology Department, Beijing Chao-Yang Hospital, Capital Medical University, Beijing, People’s Republic of China; 8 Department of Epidemiology, School of Public Health, Peking University Health Science Center, Beijing, People’s Republic of China

**Keywords:** COPD, events adjudication, COPD exacerbations, spirometry

## Abstract

**Background:**

COPD is the fourth leading cause of death worldwide, with particularly high rates in the People’s Republic of China, even among never smokers. Large population-based cohort studies should allow for reliable assessment of the determinants of diseases, which is dependent on the quality of disease diagnoses. We assessed the validity of COPD diagnoses collected through electronic health records in the People’s Republic of China.

**Methods:**

The CKB study recruited 0.5 million adults aged 30–79 years from ten diverse regions in the People’s Republic of China during the period 2004–2008. During 7 years of follow-up, 11,800 COPD cases were identified by linkage with mortality registries and the national health insurance system. We randomly selected ~10% of the reported COPD cases and then undertook an independent adjudication of retrieved hospital medical records in 1,069 cases.

**Results:**

Overall, these 1,069 cases were accrued over a 9-year period (2004–2013) involving 153 hospitals across ten regions. A diagnosis of COPD was confirmed in 911 (85%) cases, corresponding to a positive predictive value of 85% (95% confidence interval [CI]: 83%–87%), even though spirometry testing was not widely used (14%) in routine hospital care. The positive predictive value for COPD did not vary significantly by hospital ranking or calendar period, but was higher in men than women (89% vs 79%), at age ≥70 years than in younger people (88%, 95% CI: 85%–91%), and when the cases were reported from both death registry and health insurance systems (97%, 95% CI: 94%–100%). Among the remaining cases, 87 (8.1%) had other respiratory diseases (chiefly pneumonia and asthma; n=85) and 71 (6.6%) cases showed no evidence of any respiratory disease on their clinical records.

**Conclusion:**

In the People’s Republic of China, COPD diagnoses obtained from electronic health records are of good quality and suitable for large population-based studies and do not warrant systematic adjudication of all the reported cases.

## Introduction

COPD is the fourth leading cause of death worldwide.^1^ In the People’s Republic of China, COPD is the third leading cause of mortality and morbidity after cerebrovascular and ischemic heart diseases, but the disease rates vary substantially between different regions.^2^ Although the Global Initiative for Chronic Obstructive Lung Disease (GOLD) criteria for COPD,^3^ an international endeavor designed to indicate best clinical practice,^4^ has recently been adopted in the People’s Republic of China, adherence to GOLD guidelines may be suboptimal due to established patterns of clinical practice and unequal distribution of health resources in the People’s Republic of China.^4^ For example, spirometry, now required to diagnose COPD,^3^ is carried out in less than one-third of COPD cases in the People’s Republic of China and is rarely available in rural areas.^5^,^6^ Such variations in clinical practice may hamper the validity of COPD diagnoses obtained from routine clinical care, which may adversely affect observational analyses of COPD in large cohort studies such as the China Kadoorie Biobank (CKB) study.^7^

Population-based prospective cohort studies are essential to investigate the relevance of lifestyle, environmental, and genetic factors for a wide range of disease outcomes. To enable efficient and cost-effective collection and ascertainment of large number of disease outcomes, many studies are increasingly using routinely collected electronic medical records during follow-up. However, the quality of such disease outcome data may vary greatly between different countries, and between different settings within the same countries, and hence need to be carefully assessed, perhaps through independent review and adjudication of a sample of the reported disease cases to inform strategies for analyses.^8^–^10^ In many of the previous studies, the validity of COPD cases has rarely been assessed properly, and even when they did so, it was often assessed through questionnaire, self-reported diagnoses, or medical records from primary care rather than hospital settings.

The CKB is a nationwide prospective cohort study of 0.5 million adults from ten diverse Chinese regions,^11^ in which incident cases of disease outcomes (including COPD) are collected periodically through death registries, disease registries, and a newly established national health insurance (HI) system. The aims of the present report were: 1) to examine the validity of incident cases of COPD in a subset of the reported cases and 2) to identify clinical, socioeconomic, and health care system-related factors that may affect the validity of diagnosis of COPD.

## Methods

### Study design

Details of the CKB study design, procedures, and study participants have been previously described.^7^,^11^ Briefly, the baseline survey was conducted in ten geographical regions (Figure S1) chosen to include a range of behavioral, lifestyle, and environmental risk factors and disease patterns. In each region, temporary assessment clinics were set up within various local residential centers during the period 2004–2008. Individuals aged 35–74 years from 100 to 150 administrative units (rural villages or urban residential committees) in each region were invited to attend the survey clinics. Approximately, 30% responded and a total of 512,891 participants were enrolled, including a few volunteers just outside the specified age range. All participants provided written informed consent. Approvals from international (Oxford Tropical Research Ethics Committee), national (Chinese Academy of Medical Sciences), and local ethics (from ten Centers for Disease Control and Prevention [CDC] of each region) committees were obtained prior to start of the study.

#### Follow-up for mortality and morbidity

The morbidity and mortality of each participant was monitored regularly through the People’s Republic of China’s CDC Disease Surveillance Points (DSP) system, checked annually against local residential records and HI records, and by active confirmation through street committee or village administrators. Causes of death from official death certificates were reported to the local CDC and coded using the tenth International Classification of Diseases (ICD-10) by trained staff, blinded to baseline information. If necessary, information from death certificates was supplemented by a review of medical records. For four major diseases (stroke, ischemic heart diseases, diabetes, and cancer), information on incidence was also collected through linkage with existing disease registries. In addition, electronic record linkage was established with the HI system that records details of all hospital admissions (including description of diagnoses, procedures, and ICD-10 codes). All records for COPD from any source were checked and standardized. By January 1, 2014, a total of 11,799 COPD (ICD-10: J41–J44) cases were identified from various sources (Figure S2), with 87% obtained from HI records and the remainder from death registries.

### Collection of clinical information for COPD

Among the 11,799 reported cases of COPD durinĝ7 years of follow-up, we randomly selected ~10% for retrieval of medical records. In the event that the relevant medical records could not be retrieved for certain cases, especially those who were admitted to hospital many years ago, a backup list of cases was provided to ensure that at least 1,000 cases (ie, 100 cases in each of the ten regions) were adjudicated. Based on the information generated and provided centrally by the CKB coordinating centers, the medical notes were collected by trained CKB staff who visited the hospital following formal approval from local health authorities and relevant hospital administration. Electronic photographs of all relevant sections of the medical records were collected and sent to the National Coordinating Centre for review of the data completeness. Although a total of 1,138 medical records were retrieved, 69 cases were subsequently excluded as they were duplicates, leaving 1,069 cases with relevant medical records for adjudication.

### Adjudication of COPD

Following verification of completeness of data by the National Coordinating Centre, the collected medical records were sent for independent adjudication to five physicians with a working knowledge of respiratory diseases, who, in turn, were supervised by a senior consultant with specialist accreditation in respiratory diseases. Based on the medical records, the physicians then completed a specific electronic database designed on the basis of extracted information and completed a disease validation form (Figure S3) that included sections on sociodemographic, clinical, and adjudicated outcome for each case.

Although multiple medical and other related criteria help inform the diagnosis of COPD, the disease remains a clinical diagnosis and no single test result is, on its own, diagnostic for COPD. COPD cases were thus adjudicated on the basis of the clinical judgment of the respiratory physicians, blinded to any other study-related information collected. Each case was independently reviewed by one respiratory physician taking account of information collected from the following sources, where available: 1) medical history (including risk factors and respiratory symptoms such as chronic phlegm and breathlessness); 2) radiological examinations; and 3) spirometry (prebronchodilator [forced expiratory volume in 1 second {FEV_1_}/forced vital capacity {FVC} <70%]). In addition, based on the medical records, confirmed COPD cases were classified into the following subcategories: 1) chronic bronchitis, 2) emphysema, and 3) mixture of chronic bronchitis and emphysema. Similarly, the adjudication aimed to identify the actual medical condition(s) in misdiagnosed COPD cases (absence of COPD according to medical records). Finally, to ascertain the completeness of the electronic database generated by the adjudicators, ~10% of the adjudicated cases were randomly selected for central review at the Clinical Trial Service Unit (CTSU), Oxford, UK. Following the review, we observed that completeness of data acquisition was high, with 95% of the cases meeting the requirements of the adjudication process and consensus reached on the remainder following discussion.

### Statistical analysis

Baseline characteristics were compared between individuals with and without COPD events, standardized by 5-year age group, region, and sex of the overall baseline population. Positive predictive value (PPV), defined as the proportion of participants with an original diagnosis of COPD that was confirmed, was used as a direct measure of the validity of COPD diagnoses. We used SAS 9.3 (SAS Institute Inc., Cary, NC, USA) for all the statistical analyses.

## Results

Overall, relevant medical records were retrieved for 1,069 cases from 153 hospitals for adjudication, which covered a 9-year period from 2004 to 2013. Table 1 shows a comparison of the baseline characteristics of 1,069 adjudicated cases with the total of 11,799 COPD cases. Overall, the adjudicated cases had mean age, education, household income, and smoking prevalence similar to the overall COPD cases. Conversely, adjudicated cases were more likely to be urban dwellers and have lower lung function and more severe COPD, as assessed by GOLD. With the exception of ischemic heart disease prevalence, which was higher in the adjudicated cases than in all reported COPD cases, there was little difference in the reported prevalence of hypertension, stroke, and diabetes between adjudicated COPD cases and all reported cases.

**Figure 1:**
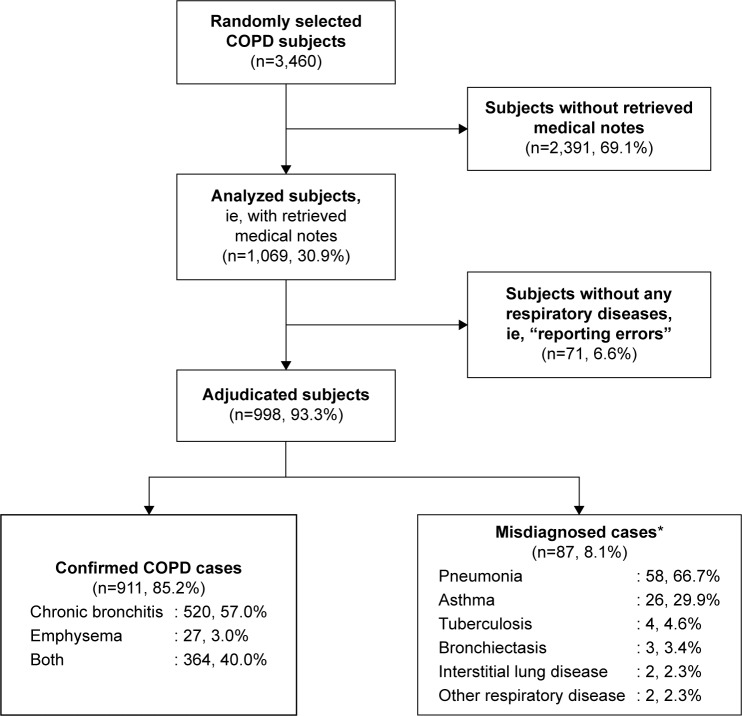
Flow diagram summarizing the process of COPD adjudication. **Note:** *Some misdiagnosed cases had multiple diseases.

Among the 1,069 cases, 71 (6.6%) had no mention of any respiratory disease in their medical records (Figure 1). In the remaining 998 cases, COPD was confirmed in 911 (85.2% of 1,069) following adjudication (Figure 1) and misdiagnosed in 87 (8.1%), as other respiratory diseases (85 cases), mainly pneumonia (58 cases and/or asthma [26 cases]), and pulmonary heart disease (2 cases). Of the 911 confirmed COPD cases, 520 had chronic bronchitis, 27 had emphysema, and the remaining 364 had both chronic bronchitis and emphysema.

The validity of COPD diagnoses, assessed by PPV, was 85% (95% confidence interval [CI]: 83%–87%) overall, higher for ICD-10 J44 (87%, 95% CI: 84%–90%), followed by J43 (85%, 95% CI: 78%–92%), and then J42 (84%, 95% CI: 81%–88%). The PPV varied across regions (heterogeneity, *I*^2^=82%, *P*<0.001) (Table 2). The PPV was 84% (95% CI: 81%–87%) when using the HI system and increased to 97% (95% CI: 94%–100%) when combined with death registries. The validity of COPD diagnoses was significantly higher in men (89%, 95% CI: 87%–92%) than in women (79%, 95% CI: 75%–84%), and in rural regions (89%, 95% CI: 86%–91%) than in urban ones (82%, 95% CI: 78%–85%). Validity of COPD was significantly higher (*P*=0.027) in Tier 2 hospital (89%, 95% CI: 85%–93%) compared to Tier 3 hospital (83%, 95% CI: 78%–86%). There was a significant positive trend (*P* for trend =0.01) for increased validity of COPD diagnoses with increasing age, with PPV of 79% (95% CI: 73%–85%), 85% (95% CI: 82%–89%), and 88% (95% CI: 85%–91%) for age groups <60 years, 60–69 years, and ≥70 years, respectively. Prebronchodilator spirometry was used in 13.9% (n=139) of the total 998 adjudicated cases, and postbronchodilator spirometry was used in only 5.7% (n=57).

## Discussion

This outcome validation study of more than 1,000 COPD cases covered 153 hospitals across ten regions, and it showed that COPD diagnoses reported through routine health record systems are of good quality in the People’s Republic of China, with an overall PPV of 85%. Invalid diagnoses arose from either misdiagnoses (~8%) of other respiratory diseases or reporting errors (~7%). The high validity of COPD diagnoses in CKB should facilitate reliable assessment of the determinants of COPD in the population.

From an international perspective, the 85% true positive estimate for COPD diagnoses in the present study is higher than that reported previously in several studies^12^–^14^ on Western populations. For example, in a Dutch study including 257 cases of chronic lung diseases from general practices in 1988, the PPV was 62.5%,^12^ similar to the 60% reported in the UK CPRD-GOLD study during 2004–2012, which involved 951 cases,^13^ or the 80% among selected 313 cases from the CPCSSN study,^14^ which was initiated in 2004. The Dutch study was conducted before the launch of the GOLD initiative, and the adjudication used a combination of pulmonary function testing and X-rays to ascertain the presence of the disease.^12^ The CPRD-GOLD study^13^ used different algorithms including, as in the present study, COPD-related clinical codes, respiratory symptoms, spirometry results, and medication use. Likewise, the Canadian study conducted in the Saskatchewan province^15^ developed case-finding diagnostic algorithms to identify cases with COPD using ICD-9 codes (490–496) from billing data, laboratory test results, and medications. The findings of the present study are lower than the 91.2% reported in the Swedish Inpatient Registry,^16^ probably due to higher and more systematic use of pre- and postbronchodilator spirometry. Interestingly, in the Canadian survey, the validity of the diagnoses varied between 64.0% and 87.7% depending on the subtype of COPD based on ICD-9 codes.^15^ These findings are consistent with the present study, with ICD-10 code J44 (including various forms of chronic and obstructive bronchitis) yielding the highest percentage of true positives followed by J42 (unspecified chronic bronchitis) and J43 (emphysema).

The difference in the reported validity of COPD diagnoses between different studies may reflect the calendar period when the disease was diagnosed and continuous improvement in diagnosis of COPD over the last few decades. The GOLD Initiative, launched in 1997, represented an important strategy to address the worldwide burden of COPD. Even before the GOLD initiative was proposed, a study^17^ of secular trends of COPD admissions in four hospitals in Barcelona over two different periods reported that the kappa values for validity of diagnosis of COPD increased from 0.20 to 0.65 between 1985–1987 and 1989. In the People’s Republic of China, GOLD guidelines were only endorsed in 2013. Therefore, the present study, which covers the period prior to 2013, is not able to address whether endorsement of the GOLD guidelines has had any measurable effect on how COPD patients are managed. In the present study, 8% of the reported COPD cases were actually due to misdiagnoses of other respiratory diseases, which reinforces that COPD is a challenging diagnosis, particularly in the early stages of the disease and when the alternative diagnosis is asthma. This is particularly true when spirometry is not widely used in many low- and middle-income countries such as the People’s Republic of China.

The validity of COPD diagnoses in the present study was 84% for cases that were solely reported through the electronic HI system, but increased to 97% when a combination of death registry and electronic HI data were used, even though the latter accounted for only a small proportion of the reported cases. Although the HI system followed common frameworks and procedures, it was developed mainly to facilitate reimbursement of hospital care, with the data collected by different HI agencies in each region lacking a uniform reporting system. This may explain some reporting errors that could have occurred either during the recording of the cases in the different regional systems or during the coding processes themselves. The HI agencies are currently endeavoring to develop a uniform and standardized reporting system, and some of the agencies have merged; therefore, administrative errors should decrease in the future.

The validity of the COPD diagnoses was slightly higher in rural regions than in the urban ones. This observation is surprising since rural health care facilities in the People’s Republic of China are less well equipped (including poor access to spirometry testing).^6^ It is possible that in rural areas COPD cases may present in more advanced stages of disease^2^ and, hence, are more easily diagnosed. In addition, our results could have been biased toward the rural regions as only 15% of the total COPD cases were from urban regions, whereaŝ37% of the adjudicated cases were from the urban areas.

There are some limitations to this study. First, the sample of adjudicated cases may not be representative of all the COPD cases in CKB or, indeed, in the People’s Republic of China. Indeed, some baseline characteristics differed between adjudicated and nonadjudicated cases. For example, lung function was lower in the sample analyzed, which could have yielded more severe cases of COPD that could have been more easily diagnosed and have had more comorbidity. This situation could reflect the fact that the medical records of participants with more hospital admissions, and consequently with more recent ones, may have been more likely to be retrieved. Second, the vast majority of COPD cases hospitalized were not assessed with spirometry, reflecting a well-recognized phenomenon of COPD management in the People’s Republic of China.

## Conclusion

In conclusion, COPD diagnoses reported through electronic HI systems in the People’s Republic of China are generally of high quality, facilitating the conduct of large-scale epidemiological investigations of determinants of COPD, and do not warrant systematic adjudication of all reported COPD cases.

## Members of the China Kadoorie Biobank collaborative group

International Steering Committee: Junshi Chen, Zhengming Chen (principal investigator), Rory Collins, Liming Li (principal investigator), Richard Peto.

International Co-ordinating Centre, Oxford: Daniel Avery, Derrick Bennett, Yumei Chang, Yiping Chen, Zhengming Chen, Robert Clarke, Huaidong Du, Xuejuan Fan, Simon Gilbert, Alex Hacker, Michael Holmes, Andri Iona, Christiana Kartsonaki, Rene Kerosi, Ling Kong, Om Kurmi, Garry Lancaster, Sarah Lewington, John McDonnell, Winnie Mei, Iona Millwood, Qunhua Nie, Jayakrishnan Radhakrishnan, Sajjad Rafiq, Paul Ryder, Sam Sansome, Dan Schmidt, Paul Sherliker, Rajani Sohoni, Iain Turnbull, Robin Walters, Jenny Wang, Lin Wang, Ling Yang, Xiaoming Yang.

National Co-ordinating Centre, Beijing: Zheng Bian, Ge Chen, Yu Guo, Bingyang Han, Can Hou, Jun Lv, Pei Pei, Shuzhen Qu, Yunlong Tan, Canqing Yu, Huiyan Zhou.

### 10 Regional Co-ordinating Centres

Qingdao Qingdao CDC: Zengchang Pang, Ruqin Gao, Shaojie Wang, Yongmei Liu, Ranran Du, Yajing Zang, Liang Cheng, Xiaocao Tian, Hua Zhang. Licang CDC: Silu Lv, Junzheng Wang, Wei Hou.

Heilongjiang Provincial CDC: Jiyuan Yin, Ge Jiang, Shumei Liu, Zhigang Pang, Xue Zhou. Nangang CDC: Liqiu Yang, Hui He, Bo Yu, Yanjie Li, Huaiyi Mu, Qinai Xu, Meiling Dou, Jiaojiao Ren.

Hainan Provincial CDC: Jianwei Du, Shanqing Wang, Ximin Hu, Hongmei Wang, Jinyan Chen, Yan Fu, Zhenwang Fu, Xiaohuan Wang, Hua Dong. Meilan CDC: Min Weng, Xiangyang Zheng, Yijun Li, Huimei Li, Chenglong Li.

Jiangsu Provincial CDC: Ming Wu, Jinyi Zhou, Ran Tao, Jie Yang. Suzhou CDC: Jie Shen, Yihe Hu, Yan Lu, Yan Gao, Liangcai Ma, Renxian Zhou, Aiyu Tang, Shuo Zhang, Jianrong Jin.

Guangxi Provincial CDC: Zhenzhu Tang, Naying Chen, Ying Huang. Liuzhou CDC: Mingqiang Li, Jinhuai Meng, Rong Pan, Qilian Jiang, Jingxin Qing, Weiyuan Zhang, Yun Liu, Liuping Wei, Liyuan Zhou, Ningyu Chen, Jun Yang, Hairong Guan.

Sichuan Provincial CDC: Xianping Wu, Ningmei Zhang, Xiaofang Chen, Xuefeng Tang. Pengzhou CDC: Guojin Luo, Jianguo Li, Xiaofang Chen, Jian Wang, Jiaqiu Liu, Qiang Sun.

Gansu Provincial CDC: Pengfei Ge, Xiaolan Ren, Caixia Dong. Maiji CDC: Hui Zhang, Enke Mao, Xiaoping Wang, Tao Wang.

Henan Provincial CDC: Guohua Liu, Baoyu Zhu, Gang Zhou, Shixian Feng, Liang Chang, Lei Fan. Huixian CDC: Yulian Gao, Tianyou He, Li Jiang, Huarong Sun, Pan He, Chen Hu, Qiannan Lv, Xukui Zhang.

Zhejiang Provincial CDC: Min Yu, Ruying Hu, Le Fang, Hao Wang. Tongxiang CDC: Yijian Qian, Chunmei Wang, Kaixue Xie, Lingli Chen, Yaxing Pan, Dongxia Pan.

Hunan Provincial CDC: Yuelong Huang, Biyun Chen, Donghui Jin, Huilin Liu, Zhongxi Fu, Qiaohua Xu. Liuyang CDC: Xin Xu, Youping Xiong, Weifang Jia, Xianzhi Li, Libo Zhang, Zhe Qiu.

## Supplementary materials

Figure S1 The location of ten survey sites in China Kadoorie Biobank (CKB).

Figure S2 Venn diagram showing the breakdown of sources for total COPD outcomes in China Kadoorie Biobank (CKB).

Figure S3 China Kadoorie Biobank (CKB) disease validation form for chronic respiratory diseases (CRD).
**Abbreviations:** OSAS, obstructive sleep apnea syndrome; IHD, ischemic heart diseases; FEV_1_/FVC, forced expiratory volume in 1 second/forced vital capacity; DLCO, diffusing capacity of the lungs for carbon monoxide; CT, computed tomography; IgE, immunoglobulin E; PHD, Pulmonary Heart Disease; ID, identification.

## Figures and Tables

**Table 1:** Baseline characteristics of participants with incident COPD

Baseline characteristics	COPD cases retrieved for adjudication^a^	COPD cases not adjudicated	All COPD cases
N	1,069	10,730	11,799
Age, years (mean ± SD)	62.9±9.2	62.4±13.9	62.4±12.8
Female (%)	46.3	50.2	50.7
Urban (%)	37.0	13.5	15.1
Follow-up time, years (mean ± SD)	3.7±1.7	4.7±6.3	4.6±4.9
Highest education completed (%)			
None/primary school	61.6	58.8	57.6
Middle/high school	34.7	36.9	38.4
College/university	3.7	4.2	4.0
Annual household income (%)			
<10,000 (Yuan)	11.6	12.2	11.8
10,000–34,000 (Yuan)	78.4	72.6	73.9
≥35,000+ (Yuan)	10.0	15.1	14.3
Smoking status (%)			
Current regular	25.2	27.7	26.9
Ex-regular	10.9	7.8	7.9
Never regular	63.9	64.6	65.1
Height, cm (mean ± SD)	156.3±6.6	156.2±9.5	156.2±8.7
BMI, kg/m^2^ (mean ± SD)	22.7±4.3	23.1±9.2	23.1±8.2
Self-reported health status (%)			
Poor	32.1	21.4	23.0
Fair	39.7	46.2	45.5
Good	17.4	21.1	19.9
Excellent	10.7	11.3	11.5
Lung function (mean ± SD)			
FEV_1_ (L)	1.46±0.65	1.81±1.47	1.80±1.47
FVC (L)	1.92±0.75	2.25±1.57	2.25±1.49
FEV_1_/FVC (%)	74.8±14.5	78.9±24.5	78.6±24.4
COPD severity^b^ (%)			
Grade 1–2	7.3	5.0	6.1
Grade 3	3.1	2.4	2.5
Grade 4	7.9	6.9	6.4
Grade 5	21.4	10.8	11.9
Self-reported comorbidity (%)			
Hypertension	12.0	11.9	11.5
IHD	6.2	4.0	4.1
Diabetes	2.9	2.7	2.7
Stroke/TIA	1.4	1.7	1.7

**Notes:**

a Standardized for age, sex, and regions;

b data from baseline lung function (2004–2008): COPD Grade 1–2= FEV_1_/FVC <LLN and (*z*-score of FEV_1_ −2.5 to 1.0); Grade 3= FEV_1_/FVC <LLN and (*z*-score of FEV_1_ −3.0 to −2.5); Grade 4= FEV_1_/FVC <LLN and (*z*-score of FEV_1_ −3.5 to −3.0); Grade 5= FEV_1_/FVC <LLN and (*z*-score of FEV_1_ <−3.5); and *P*-values between two groups for all characteristics are <0.05. Eventually, medical records of 1,069 participants were retrieved for adjudication.

**Abbreviations:** SD, standard deviation; BMI, body mass index; FEV_1_, forced expiratory volume in 1 second; FVC, forced vital capacity; IHD, ischemic heart diseases; TIA, transient ischemic attack; LLN, lower limit of normal.

**Table 2:** Distribution of refuted and confirmed COPD cases by regions, hospital ranking, reporting sources, sex, and age

	Number	Refuted cases n (%)	Confirmed COPD cases n (%)	aPPV % (95% CI)
Total (N)	1,069	71 (6.6)	911 (85.2)	85 (83–87)
Reporting source				
HI only	961	71 (7.4)	806 (83.9)	84 (81–87)
Death registry and HI	108	0 (0.0)	105 (97.2)	97 (94–100)
Hospital tier^b^				
Top rank (Tier 3)	365	25 (6.8)	303 (83.0)	83 (78–86)
Medium rank (Tier 2)	290	9 (3.1)	259 (89.3)	89 (85–93)
Low rank (Tier 1)	349	27 (7.7)	299 (85.7)	86 (83–91)
Admission year				
2004–2007	104	6 (5.8)	92 (88.5)	88 (82–95)
2008	187	8 (4.3)	168 (89.8)	90 (85–94)
2009	228	15 (6.6)	191 (83.8)	84 (78–89)
2010	231	19 (8.2)	190 (82.3)	82 (77–88)
2011–2013	319	23 (7.2)	270 (84.6)	85 (80–89)
Sex				
Male	611	23 (3.8)	547 (89.5)	89 (87–92)
Female	458	48 (10.5)	364 (79.5)	79 (75–84)
Age groups (in years)				
<60	210	23 (11.0)	166 (79.0)	79 (73–85)
60–69	397	22 (5.5)	339 (85.4)	85 (82–89)
70+	462	26 (5.6)	406 (87.9)	88 (85–91)
Regions				
Urban	531	35 (6.6)	434 (81.7)	82 (78–85)
Qingdao	120	3 (2.5)	105 (87.5)	87 (81–94)
Harbin	123	3 (2.4)	100 (81.3)	81 (74–89)
Haikou	40	3 (7.5)	37 (92.5)	92 (84–100)
Suzhou	125	8 (6.4)	106 (84.8)	85 (78–92)
Liuzhou	123	18 (14.6)	86 (69.9)	70 (60–80)
Rural	538	36 (6.7)	477 (88.7)	89 (86–91)
Sichuan	117	4 (3.4)	110 (94.0)	94 (90–98)
Gansu	79	13 (16.5)	58 (73.4)	73 (62–85)
Henan	113	15 (13.3)	93 (82.3)	82 (74–90)
Zhejiang	115	3 (2.5)	108 (93.9)	94 (89–98)
Hunan	114	1 (0.9)	108 (94.7)	95 (91–99)

**Notes:**

a Positive predictive value (subjects with confirmed COPD cases/total subjects selected for COPD adjudication) rounded up to no decimal place;

b hospital tier not available for 65 cases. Out of total 1,069 cases, 71 were refuted cases with no respiratory diagnoses and 87 cases were misdiagnosed as CODP although they were other respiratory diseases.

**Abbreviations:** PPV, positive predictive value; HI, health insurance; CI, confidence interval.
