# Supplementary figures and images for: Validity of COPD diagnoses reported through nationwide health insurance systems in the People’s Republic of China
Source: Int J Chron Obstruct Pulmon Dis. 2016 Mar 1;11:419–30. doi: 10.2147/COPD.S100736 (PMC4780206; doi:10.2147/COPD.S100736)

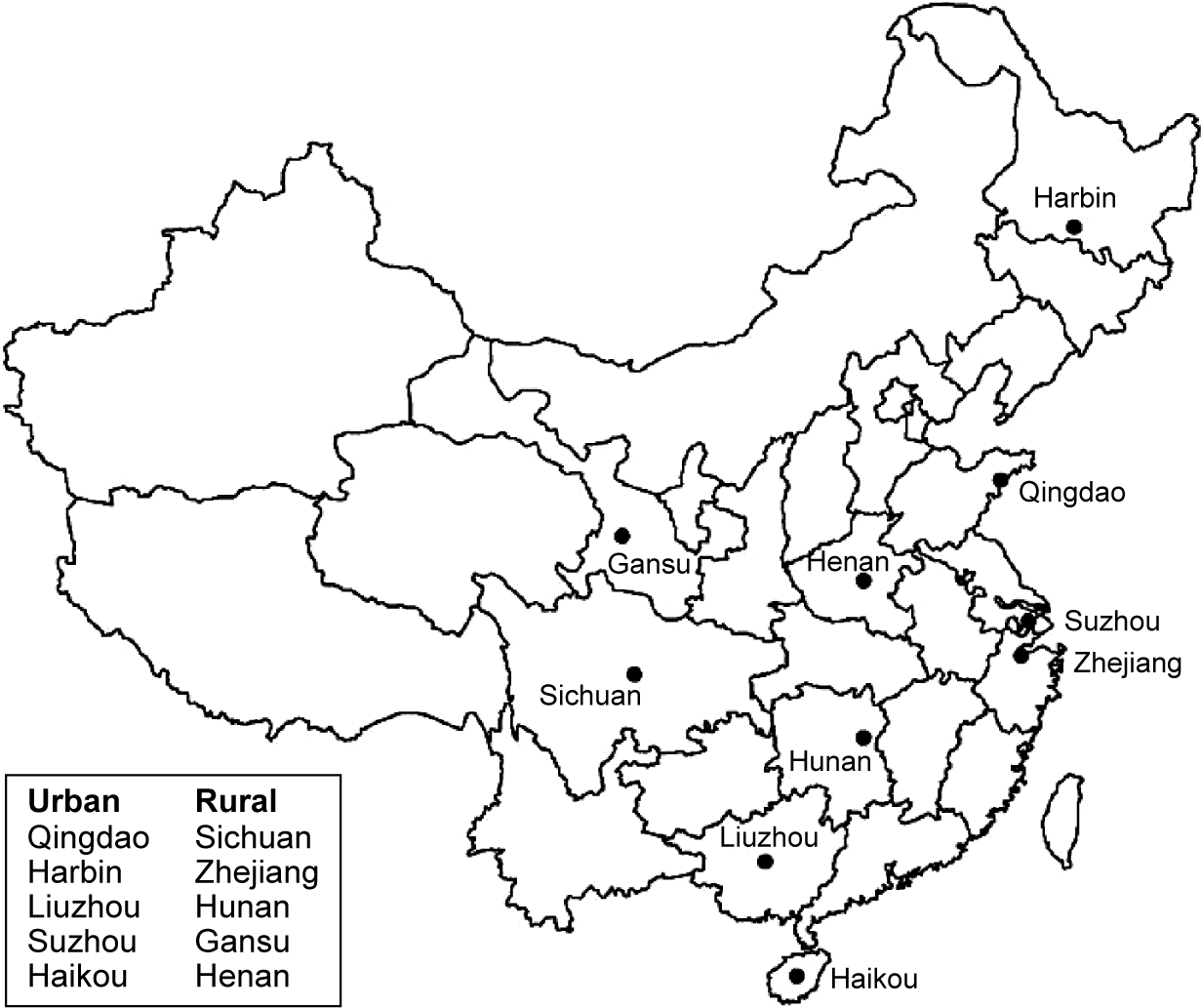

Supplement: Figure S1 — The location of ten survey sites in China Kadoorie Biobank (CKB). [file copd-11-419s1.tif]

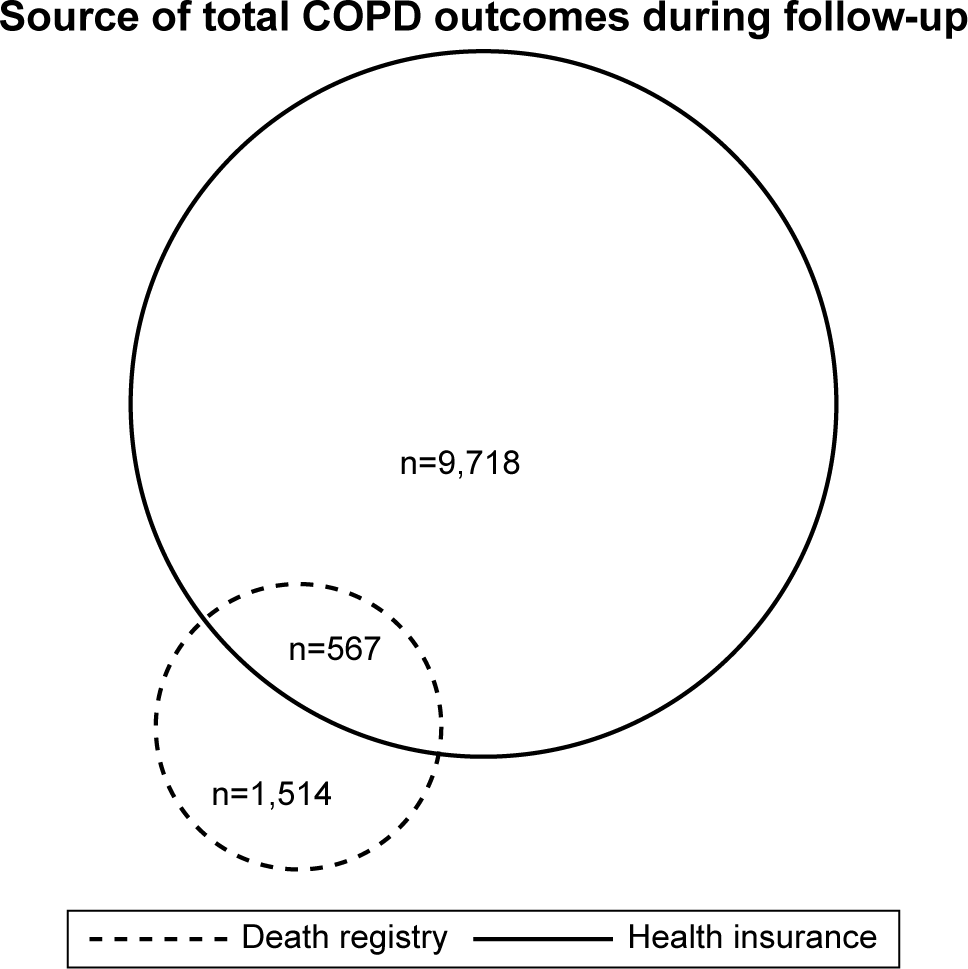

Supplement: Figure S2 — Venn diagram showing the breakdown of sources for total COPD outcomes in China Kadoorie Biobank (CKB). [file copd-11-419s2.tif]

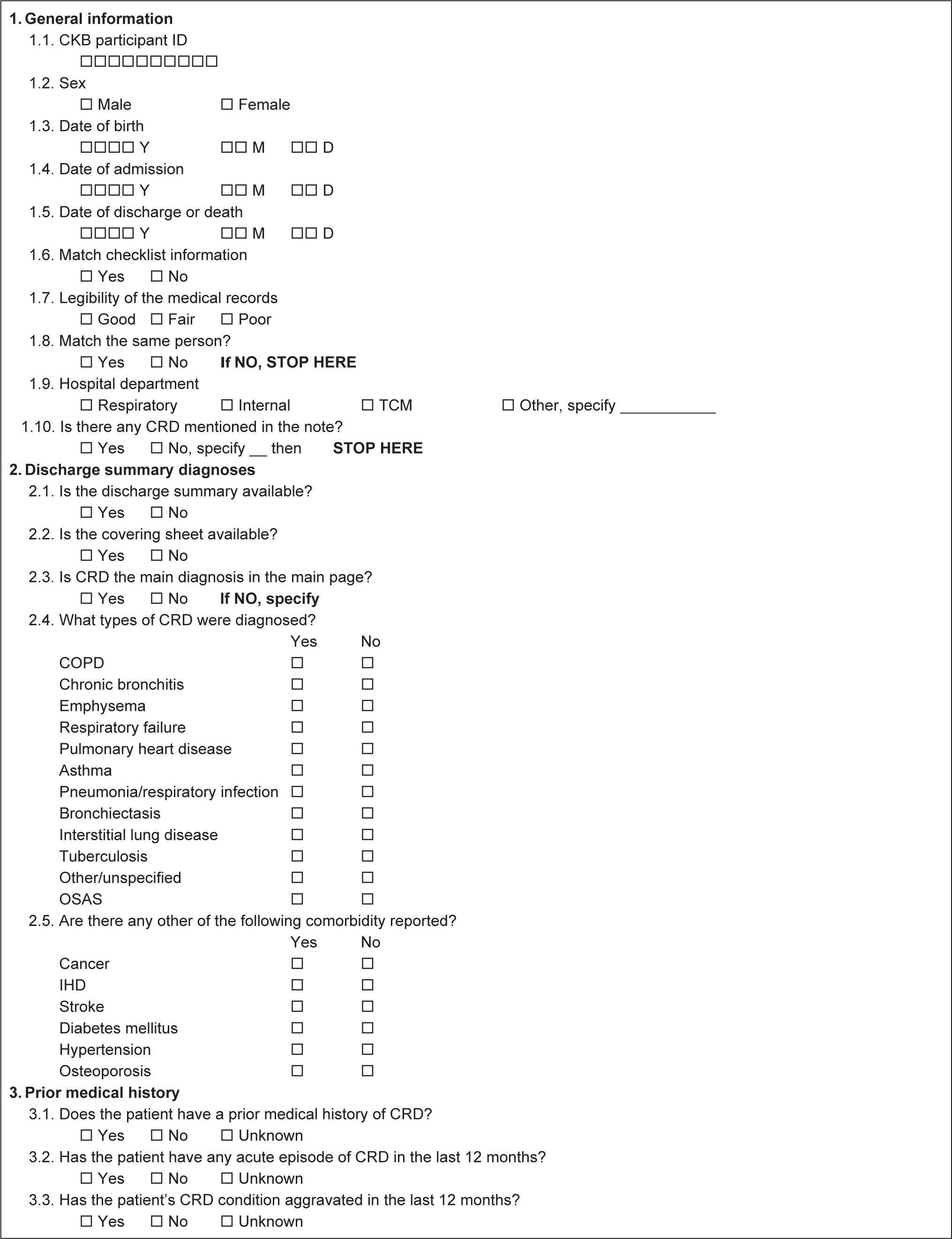

Supplement: Figure S3 — China Kadoorie Biobank (CKB) disease validation form for chronic respiratory diseases (CRD). Abbreviations: OSAS, obstructive sleep apnea syndrome; IHD, ischemic heart diseases; FEV1/FVC, forced expiratory volume in 1 second/forced vital capacity; DLCO, diffusing capacity of the lungs for carbon monoxide; CT, computed tomography; IgE, immunoglobulin E; PHD, Pulmonary Heart Disease; ID, identification. [file copd-11-419s3.tif]

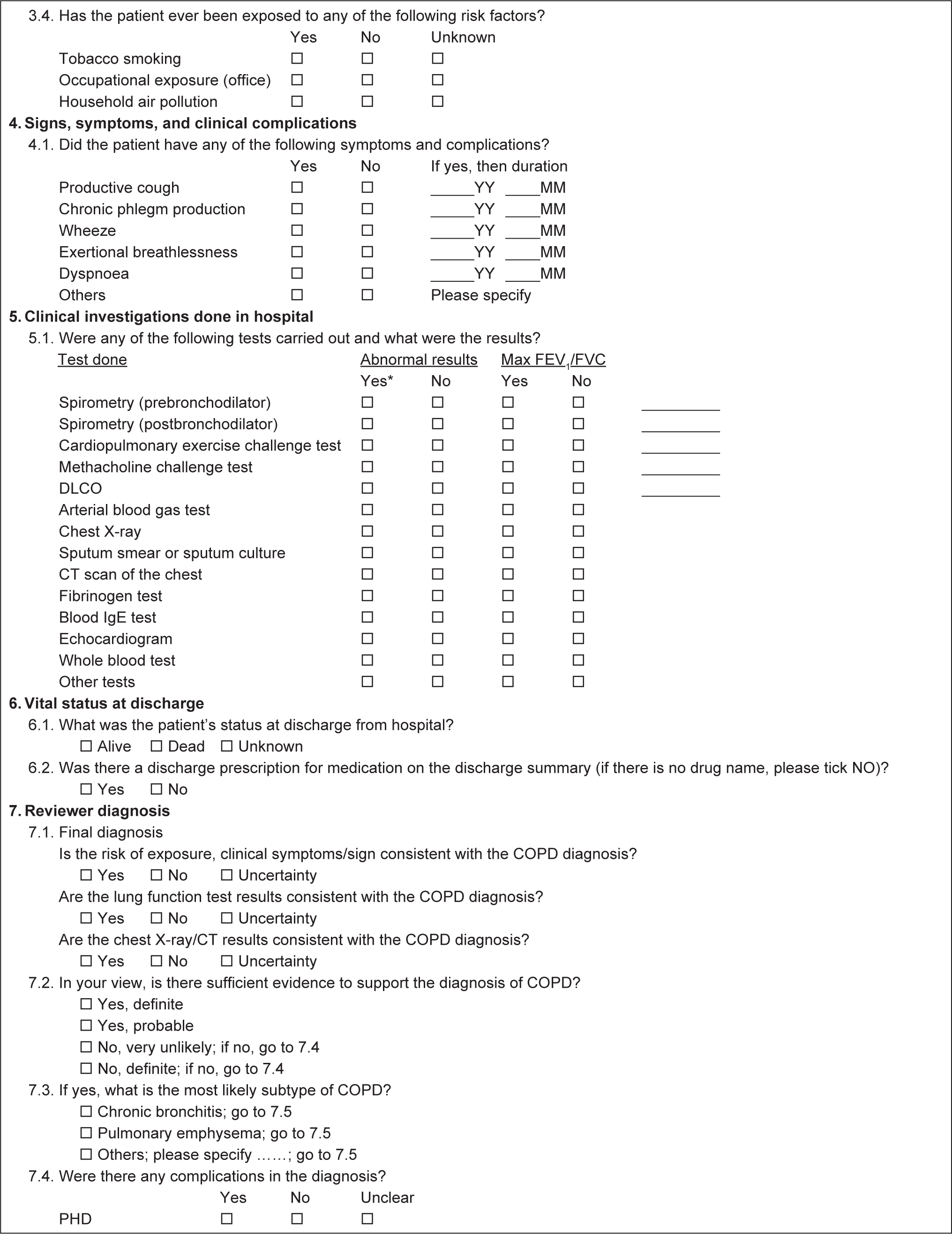

Supplement: Figure S3 — China Kadoorie Biobank (CKB) disease validation form for chronic respiratory diseases (CRD). Abbreviations: OSAS, obstructive sleep apnea syndrome; IHD, ischemic heart diseases; FEV1/FVC, forced expiratory volume in 1 second/forced vital capacity; DLCO, diffusing capacity of the lungs for carbon monoxide; CT, computed tomography; IgE, immunoglobulin E; PHD, Pulmonary Heart Disease; ID, identification. [file copd-11-419s3a.tif]

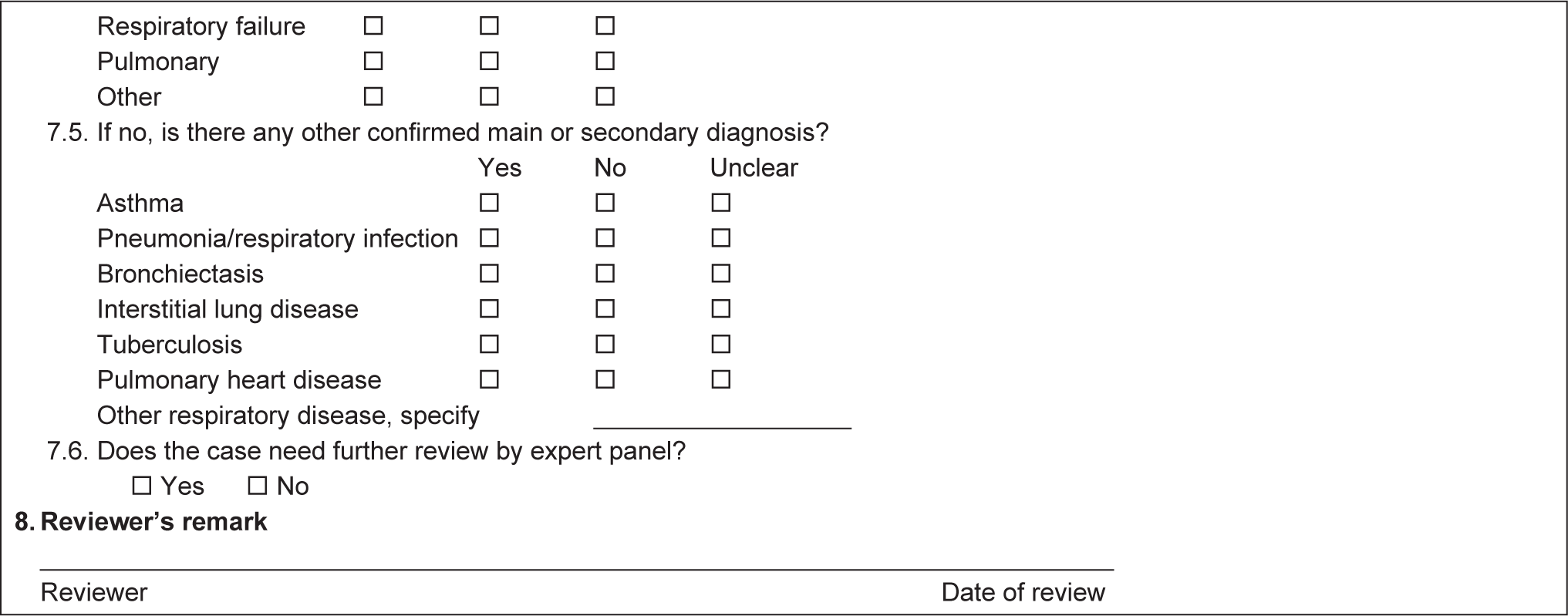

Supplement: Figure S3 — China Kadoorie Biobank (CKB) disease validation form for chronic respiratory diseases (CRD). Abbreviations: OSAS, obstructive sleep apnea syndrome; IHD, ischemic heart diseases; FEV1/FVC, forced expiratory volume in 1 second/forced vital capacity; DLCO, diffusing capacity of the lungs for carbon monoxide; CT, computed tomography; IgE, immunoglobulin E; PHD, Pulmonary Heart Disease; ID, identification. [file copd-11-419s3b.tif]
